# Longitudinal study of *Plasmodium* pathogens identifies new loci associated with artemisinin resistance

**DOI:** 10.1186/s13059-017-1219-x

**Published:** 2017-04-28

**Authors:** Elizabeth A. Winzeler

**Affiliations:** Department of Pediatrics, University of California, San Diego, La Jolla, California 92093 USA

## Abstract

A longitudinal analysis of malaria parasite genomes has revealed new markers that can be used in public health efforts to limit the spread of multidrug-resistant malaria.

Malaria is a devastating tropical disease that can be fatal if untreated. It is caused by protozoan pathogens of the genus *Plasmodium* and is transmitted by *Anopheles* mosquitoes. Up to 40% of the world’s population lives in areas where malaria exists and, despite progress, there were still 214 million cases of malaria and 438,000 deaths in 2015. Owing to the huge burden caused by this disease, studies on the treatment resistance of malarial parasites are essential and a recent longitudinal study by Cerqueira and colleagues, published in *Genome Biology* [1], is an important contribution.

## Controlling malaria

Given that there is no sterilizing immunity and no highly effective vaccine that can be used against malaria, this disease is mostly controlled with bed nets, insecticide spraying, and chemotherapies such as artemisinin combination therapies (ACT). ACTs combine new endoperoxide-type compounds (such as artemether or artesunate) with older antimalarial drug classes, such as an aryl alcohol (lumefantrine) or an aminoquinoline (e.g., piperaquine), and provide rapid symptomatic relief. The World Health Organization currently recommends five ACTs, including artemether–lumefantrine, artesunate–amodiaquine, artesunate–mefloquine, artesunate–sulfadoxine–pyrimethamine (ASSP), and dihydroartemisinin–piperaquine. Unfortunately, malaria parasites have known resistance to older antimalarial drugs, which stems from the historical use of these drugs as a monotherapy or even as a folk remedy. For example, aminoquinolines work by the same mechanism as quinine, which is the active ingredient in antimalarial tonic water and Jesuit’s bark. Although new types of antimalarial drugs are in development (https://www.mmv.org/research-development/mmv-supported-projects), their slow progress to the clinic means that current ACT developers need to select partner drugs from a limited set of older antimalarial compounds.

## Artemisinin resistance

**Fig. 1:**
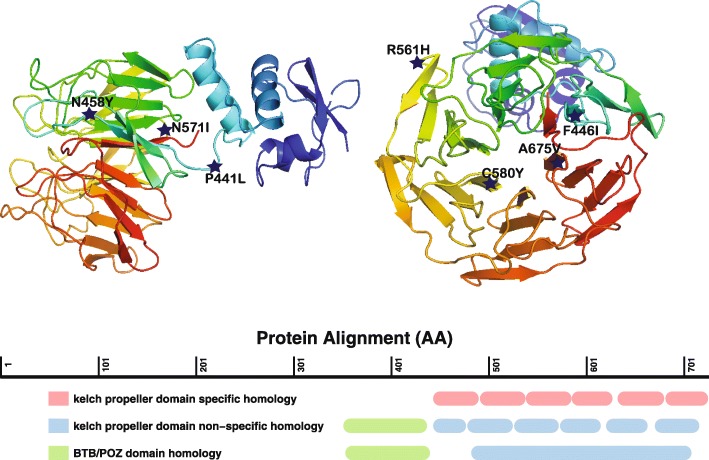
Homology model of the Kelch13 protein showing the positions of mutations detected in the study in a side view (*left*) and frontal view (*right*). Mutations are located in the Kelch domain, in some cases near the central cavity (C580Y and A675Y) that is involved in protein binding. The model was constructed using SwissModel and pdb template 4yy8.1.A

Owing to the weaknesses of the older partner drug classes, successful combination therapies depend on the endoperoxide component. Thus, when parasites that are resistant to artemisinin monotherapy first began to appear in Southeast Asia in 2008, a worldwide effort was initiated to find the genes that are associated with artemisinin resistance in order to study and contain the spread of resistance. The causative gene, *kelch13*, encoding an uncharacterized protein with kelch- and BTB/POZ-propeller domains (Fig. 1), was identified using a combination of genome-wide association studies (GWAS) [2–4] and laboratory-based in vitro evolution [2]. Although the mechanism by which *kelch13* mutations confer resistance remains poorly understood, the introduction of *kelch13* single-nucleotide variants (SNVs) into an artemisinin-sensitive parasite line by genome editing results in artemisinin-sensitive parasites [5].

Despite these advances, the community has continued to wonder whether mutations in *kelch13* are the only determinants of resistance, especially as genome-wide association tests have shown that other parasite loci are associated with resistance and delayed parasite clearance [4] in Southeast Asia. In addition, there are questions as to why the *kelch13* mutations, which seem to have arisen multiple independent times, are found primarily in parasites in Southeast Asia.

## Discovering markers of resistance

To address these questions, an international team led by Daniel Neafsey at the Broad Institute used a new approach. Previous GWAS had used samples that were collected primarily after resistance had emerged, but the Neafsey team performed a longitudinal analysis, sequencing 194 parasites from patient samples obtained from five sites in Northwest Thailand during the period in which artemisinin resistance emerged (2001–2014) [1]. They first identified approximately 17,000 high-quality SNVs. Then, using artemisinin-resistance data (parasite clearance time) as a phenotype, they performed a GWAS. The authors showed that, as expected, the strongest association signal was from *kelch13*, with parasites bearing the C580Y mutant allele dominating by the end of the surveillance period. These data beautifully show that GWAS can indeed find a specific allele and not just important loci.

Next, the authors sought to find potential modulatory alleles that may have supported the emergence of *kelch13* mutant alleles. Here, the data were reanalyzed with only samples containing mutant *kelch13* alleles included. Interestingly, this GWAS analysis identified another variant in a kelch-domain-containing gene on chromosome 10, which suggests that variants in this gene, *kelch10*, may epistatically modulate the artemisinin-resistance phenotype.

Finally, the authors performed a longitudinal analysis to identify candidate background mutations that were required for the spread of *kelch13* resistance mutations. Unlike other significant variants that were identified in the GWAS study, *kelch13* variants exhibited a temporal increase in the frequency of the non-reference allele (C580Y) during the period when artemisinin resistance evolved. To identify other genes that might contribute to resistance, the authors further analyzed their dataset and identified other variants that, like *kelch13* variants, show an increase in non-reference allele frequency over time. Notably, some of these variants occur in genes that belong to pathways thought to be involved with artemisinin resistance in *Plasmodium falciparum*, including the phosphoinositol signaling pathway [6] and the ubiquitination pathway [7]. The authors hypothesize that these variants may increase the fitness of parasites that have the *kelch13* gene.

## Future work

A potential weakness in Cerqueira et al.'s analysis [1] is that it focused entirely on SNVs and did not consider structural variants. Recently, researchers have shown that copy number variants on chromosome 14 are associated with resistance to piperaquine, a component of an ACT used in Southeast Asia [8, 9]. In addition, copy number changes in *mdr1*, which encodes the *P. falciparum* multidrug resistance protein 1, confer resistance to mefloquine, another partner drug. It is possible that important structural variants may be located near the alleles that were identified in this study as being associated with delayed parasite clearance, and that the structural variants are actually responsible for the association signal. This possibility highlights the importance of obtaining independent evidence (from genome-editing studies, for example) to confirm causation for alleles discovered in association studies.

In addition, the group was unable to confirm the role of variants that have been identified by others. A similar whole-genome study by Miotto et al. [10] using 1612 patient samples from 15 locations in Cambodia, Vietnam, Laos, Thailand, Myanmar, and Bangladesh showed that nonsynonymous variants in *fd* (*ferredoxin*), *arps10* (*apicoplast ribosomal protein S10*), *mdr2* (*multidrug resistance protein 2*), and *crt* (*chloroquine resistance transporter*) were associated with artemisinin resistance. Cerqueira and colleagues [1] could not confirm the role of these variants, perhaps because the parasites in Western Thailand are different from those that have been studied by the Miotto group. It is also worth noting that different partner drugs are used in different countries in Southeast Asia.

## What does this mean for malaria control?

The study provides immediate benefits to patients. Bacterial drug-sensitivity assays are routinely performed in clinics throughout the world, but assessing whether a patient has an artemisinin-resistant malaria infection is much more challenging. Parasites from patients often do not adapt to in vitro cell culture, and even if they do, the resistance phenotype can be subtle and very difficult to quantify. The new *kelch13* alleles identified in this study can now be incorporated into PCR-based tests that are much easier and less expensive to perform than existing tests. The results of such PCR tests can then be used to further guide therapy practices, for example, informing the duration or concentration of drug treatments. Knowledge of new resistance-conferring alleles will also help with surveillance. If ongoing genomic surveillance shows that the alleles are appearing in a new geographical region, more aggressive use of insecticides and bed nets would be warranted.

The importance of limiting artemisinin resistance in Southeast Asia cannot be underestimated, especially now that resistance to partner drugs has emerged. If the resistance alleles were to remain confined to Southeast Asia, a great increase in morbidity and mortality would be avoided. In fact, the World Health Organization has proposed attempts to eliminate malaria in this region in order to contain resistance. Luckily, the vast majority of malaria cases are in Sub-Saharan Africa, where clinical trials are showing that ACTs remain effective for now.

While Cerqueira et al.'s study [1] will be of most interest to malaria researchers, physicians, and patients, it will also be of broader interest to those outside of the field because it demonstrates how whole-genome sequencing, GWAS, and longitudinal studies can be used to provide an understanding of emerging drug resistance in eukaryotic pathogens and to identify causative alleles precisely.

## Abbreviations

ACT: artemisinin combination therapies
GWAS: genome-wide association studies
SNV: single-nucleotide variant
